# Training and performance measures for novices to the area of fingerprint analysis

**DOI:** 10.1016/j.dib.2017.06.036

**Published:** 2017-06-28

**Authors:** Sarah V. Stevenage, Alice Bennett, Christy Pitfield

**Affiliations:** Department of Psychology, University of Southampton, UK

**Keywords:** Fingerprint analysis, Training, Cognitive bias

## Abstract

The data accessible here represents the training tool used with novice students prior to an assessment of their performance in a fingerprint matching task (Stevenage and Pitfield, 2016; Stevenage and Bennett, in preparation) [1,2]. The training tool was compiled following semi-structured interviews with practicing fingerprint experts within the UK (Stevenage and Pitfield, 2016) [1], and has been verified as being a useful summary document by a subset of these experts. Also provided are the performance data of trained student participants on a fingerprint matching task. This was undertaken under biased and unbiased contextual conditions, and under control conditions in which no contextual information was provided. These resources are provided here to enable the interested reader to extend analysis in this area through studying the performance of non-naïve participants, and to complete a meta-analysis across relevant studies.

## Specifications Table

**Table:** 

Subject area	Psychology
More specific subject area	Fingerprint Analysis
Type of data	Word Document, Excel file
How data were acquired	Qualitative analysis of responses to semi-structured interviews with practicing fingerprint experts.
	Computer-based assessment of performance on a fingerprint matching task. All data were recorded using SuperLab 4.5 running on a Hewlett Packard Pavilion G Series laptop with a 15” colour monitor and a screen resolution of 1366×768 pixels.
Data format	Analysed
Experimental factors	The fingerprint training tool was developed by extracting the quotes and corresponding fingerprint images as 12 practicing UK fingerprint analysts narrated their task of fingerprint analysis.
	The performance measures were obtained from three trained groups of student participants who completed a fingerprint matching task under conditions of no time pressure (groups 1 and 3) or time pressure (group 2), and under conditions in which biased or unbiased contextual information was introduced (Groups 1 and 2) or not (Group 3).
Experimental features	A computer-based fingerprint matching task was presented to all participants in which they saw two fingerprints presented simultaneously. Their task was to report on whether the prints belonged to the same person (identification decision) or not (exclusion decision).
	Accuracy of response was calculated relative to ground truth. From this, performance on matching trials, and non-matching trials was calculated, along with a measure of response bias (C) which represented the tendency to give one answer over the other answer.
Data source location	Southampton, Hampshire, UK
Data accessibility	Data are provided in this article.

## Value of the data

• The provision of the training tool will enable other researchers to train student participants in the task of fingerprint analysis such that they can represent a non-naïve (though non-expert) population for testing.
• The data provided here offer value in allowing meta-analysis of performance on a fingerprint matching task under conditions of biased (and unbiased) contextual information.
• The data provided here offer further value in supporting analysis of the effect of *any* contextual information versus the effect of no contextual information.
• The data finally may provide value given the potential to align direction of bias with the ground truth of each fingerprint trial. This may support future analysis on a new type of ‘bias danger zone’ in which exclusion decisions are more bias-able than identification decisions.

## 1 Data

The data include the training tool for use with initially naïve student participants. In addition, performance measures are provided following training detailing accuracy on a fingerprint matching task for matching and non-matching pairs of prints, and under conditions of biased or unbiased contextual information.

## 2 Experimental design, materials and methods

The fingerprint training tool was developed following semi-structured interviews with 12 practicing fingerprint analysts at a UK fingerprint bureau [1]. Each had described the characteristics that they look for in a fingerprint, and had narrated their process of analysis, comparison and evaluation with reference to a pair of prints. Quotes were extracted from these interviews to illustrate the various fingerprint characteristics, and the process taken to reach an identification, exclusion or inconclusive decision. These quotes were used as the basis for the fingerprint training tool, and were accompanied by annotated images of the fingerprint pair that the experts had been referring to. The annotations were added merely to highlight the characteristics being discussed at each point in the training.

The training tool was used with student participants as a way of raising performance from an initially naïve starting point. Following training, and a short practice phase with feedback, performance was assessed on a same/different fingerprint matching task which featured fingerprint images drawn from the FVC2004 database. These fingerprints were represented by a good quality image (to simulate the custody suite image) and by a poorer quality image (to simulate the latent print from the crime scene). As such, matching pairs of prints never involved the presentation of identical images to avoid the task being based on image-related features rather than fingerprint-related features. Similarly, foil prints used in the non-matching trials were always selected to be of the same fingerprint pattern (ulnar loop, radial loop, whorl) so that the task was not trivially easy.

Stimuli were presented and data recorded via computer software. Participants completed 72 fingerprint matching trials (half ‘matching’, half ‘non-matching’), with order of trials randomized. Critically, trials were accompanied by case details summarizing the findings from a DNA test in the case. Those findings either suggested that the suspect was a match, or was not a match, to the perpetrator. The performance data summarized the effect of this manipulation on the accuracy of responses to matching and not-matching fingerprint trials relative to a control condition in which the DNA test result did not introduce any directional context. Full details of stimuli and procedure are provided in [2].
