# Supplementary material for: Training and performance measures for novices to the area of fingerprint analysis
Source: Data Brief. 2017 Jun 28;13:641–3. doi: 10.1016/j.dib.2017.06.036 (PMC5502696; doi:10.1016/j.dib.2017.06.036)
Supplement: Supplementary file 3 — Supplementary material [file mmc3.pdf]

FINGERPRINT TRAINING TOOL

Sarah V Stevenage and Christy Pitfield
University of Southampton

To develop essential basic understanding of fingerprint features and ACE-V protocols.

Contents Page

Introduction 2

Summary of Fingerprint Characteristics

First Level Details

Fingerprint ridge patterns 3-4

Cores and Deltas 4-5

Second Level Details

Bifurcations and Ridge Endings 6

Third Level Details

Sweat Pores 7

Creases and Scars 7-8

Analysis, Comparison, Evaluation and Verification (ACE-V) procedure

Analysis 9

Comparison 9-11

Evaluation 12

(Note: Verification is not covered here as this goes beyond the task of the first individual)

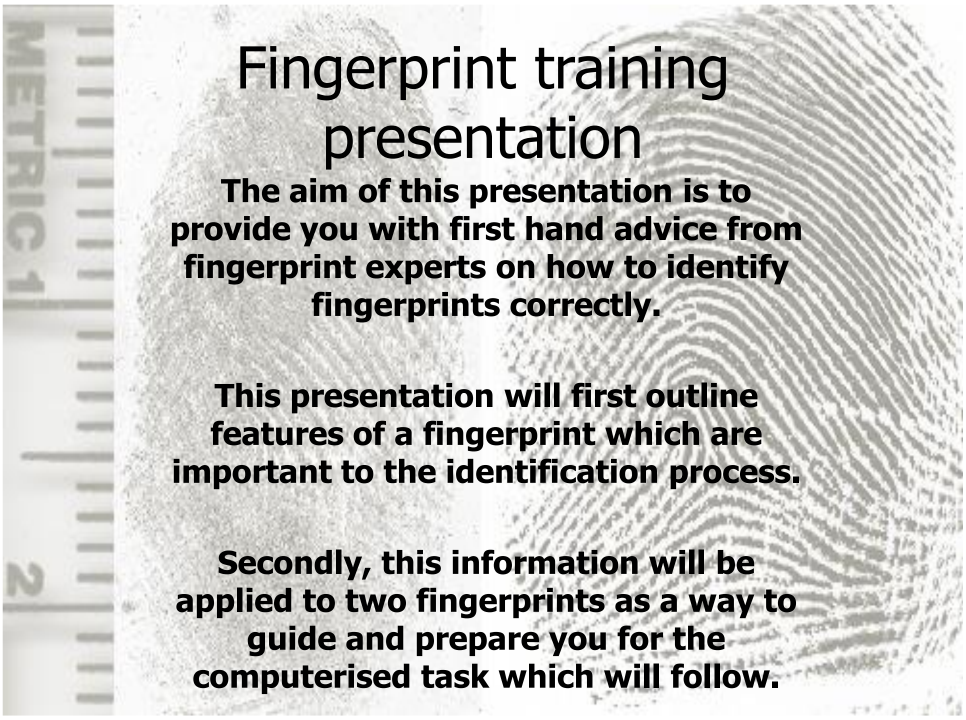
Fingerprint training presentation
The aim of this presentation is to provide you with first hand advice from fingerprint experts on how to identify fingerprints correctly.
This presentation will first outline features of a fingerprint which are important to the identification process.
Secondly, this information will be applied to two fingerprints as a way to guide and prepare you for the computerised task which will follow.

First Level Detail – Patterns

"The first thing you would look for would be what we call first level detail which is the pattern, so whether [it is] a loop or whorl or an arch."

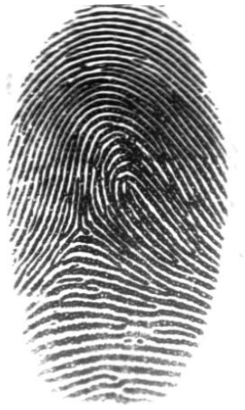

Loop

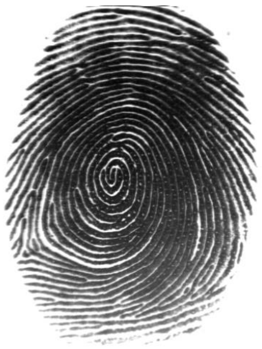

Whorl

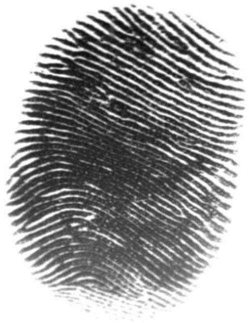

Arch

Disagreement of Pattern

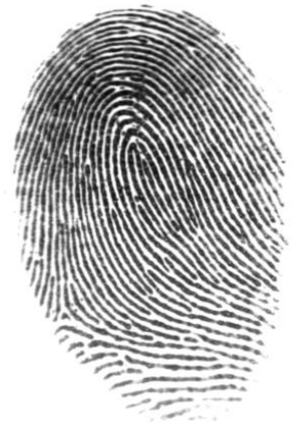

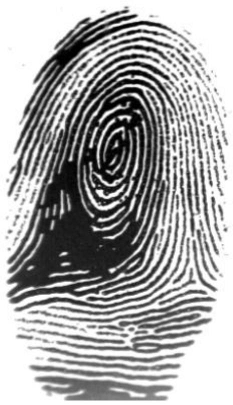

"Straight away you can see that the patterns are completely different so, to be honest, if I had [the left] as the scene of crime mark and [the right]as the suspect, straight away I could say no its not them just based on the pattern, I wouldn't even look any further into it because the pattern is obviously not the same."

Using the Pattern

How does this help?
"They help …. primarily with narrowing down where I look"

Direction:

"It would have to be a pattern in the same direction"

In the example to the left, the Loop patterns are flowing in the opposite direction so we have a non match.

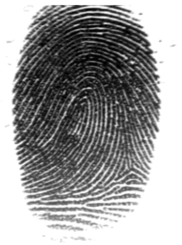

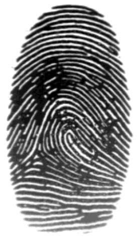

Size:

"But if [the mark is] a loop and that [individual] has all loops then also of the size of the loop helps."

"So if your crime scene mark for example was a loop and your suspects only had whorl patterns then straight away you could say that's negative, it's not identified."

Using the Pattern

"When we are looking at a fingerprint you are drawn to the pattern straight away but you have got two areas [to] which your eye is drawn."
"The first is the core, that's exactly where the pattern is."

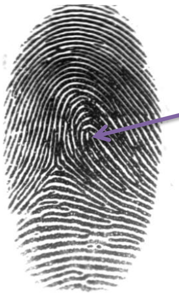

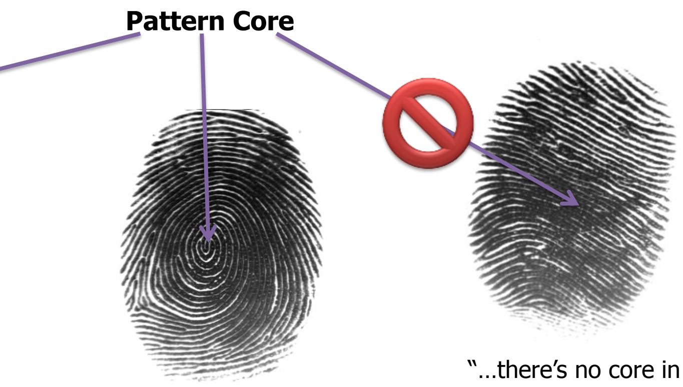

"My eye is immediately drawn to the centre of the pattern which is the core and you can see that it's like a hairpin, the ridges run round and it forms like a sort of curve shape. "

"…there's no core in an arch... they run from side to side and that's it- there's no main pattern area."

Deltas...

"where the ridges split into three"
"which are the little triangular area"

"On whorls you have two delta areas, on loops you only have the one...."

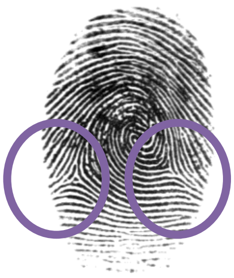

Whorl= 2

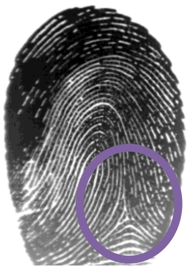

Loop= 1

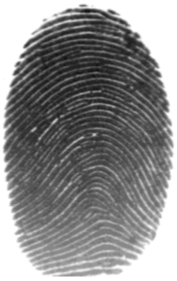

Arch= 0

Delta & Core- analyse these together

" Look to see the distance between the centre of the pattern...(the core) and also the delta"

"If you were to put a straight line from core to the point where the delta is, count the number of black lines intervening between those two points"

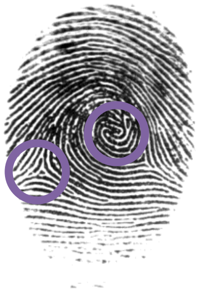

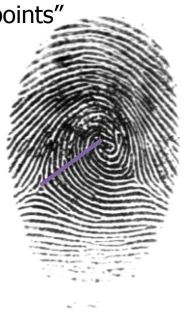

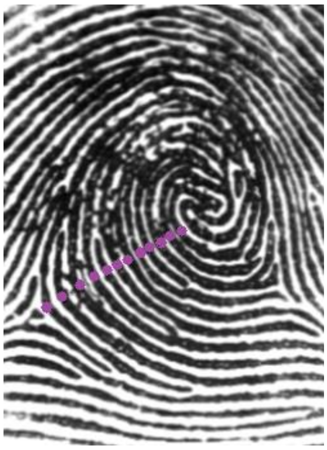

Second Level Detail

"once you've established that the pattern is the same [and the] distance between the core and delta is the same, you can look at second level detail which is the ridge endings and bifurcations"

So ridge ending would be where the ridge runs along and stops. And the bifurcation is where it would split into two separate ridges.

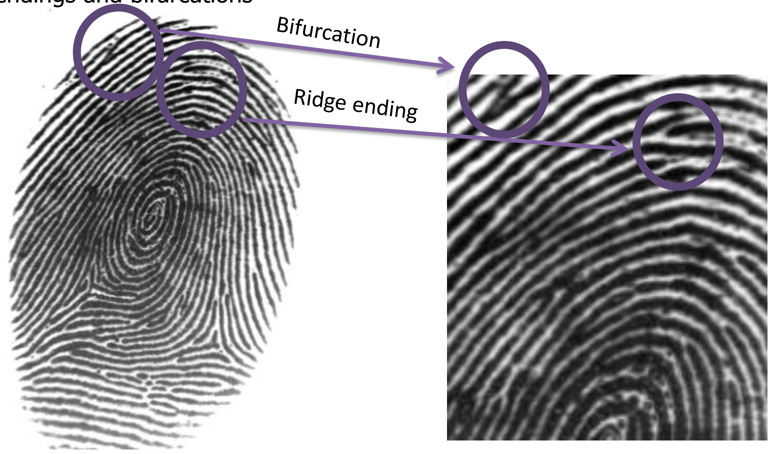

Second Level Detail

"we try to find the same types of ridge ending and bifurcations in the same place, in the same order"

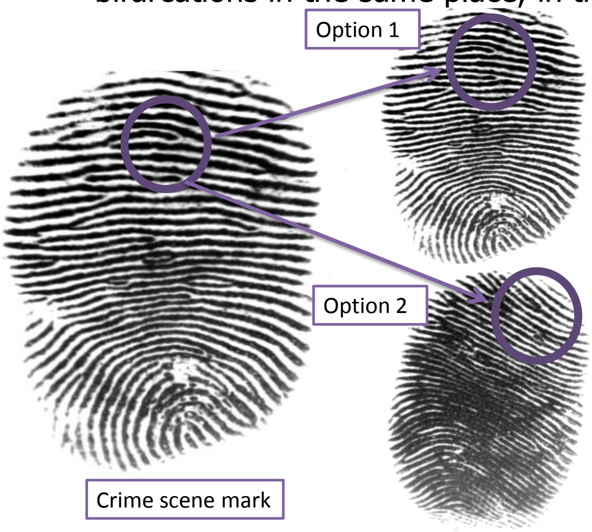

✓Both finger print options have a clear bifurcation

X Only option 1 is in the same place as the crime scene mark

•Option 1 is therefore the correct match

Third Level Detail

"....is looking at individual ridge units perhaps or looking at the relative positions of pores, (which would be poreoscopy) and/or the shape of the edges of the ridge...(something called edgeoscopy) which we don't do very much of because usually we can make an identification based on the pattern and the ridge endings and bifurcations..."

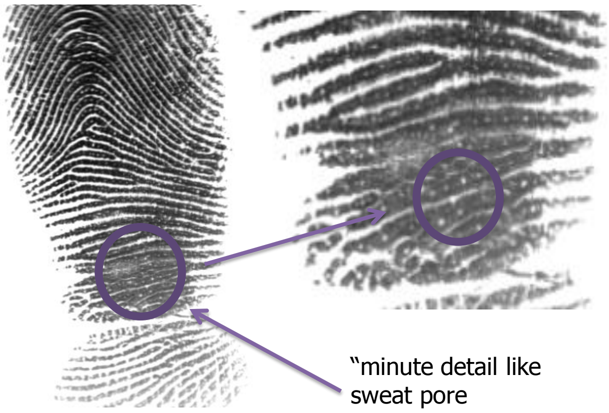

"minute detail like sweat pore locations"

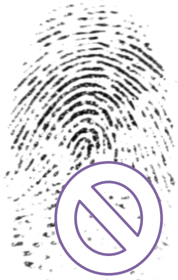

"However, whether or not people see it with enough clarity can be very dependable, it would be need to be a good quality impression to be actually able to really pluck that information."

Creases

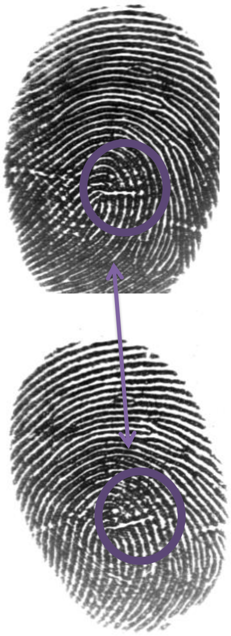

"But creases, once they're there, they will stay there, and you can use creases as part of an identification. Especially if you haven't got a lot of ridge detail, you can incorporate the use of the creases as well, and where they are – they're not going to move, they're not going to change, so you can use those as well."

"Creases… do tend to run vertical.."

Scars

"If there's scar visible on the scene of crime mark and also the fingerprint form… you can use that as a unique feature . It can be quite misleading if they just have a cut which over time heals itself- like a paper cut for example."

"If they appear in the crime scene mark and the fingerprint form you can use that as a feature to help you reach your decision on identification "

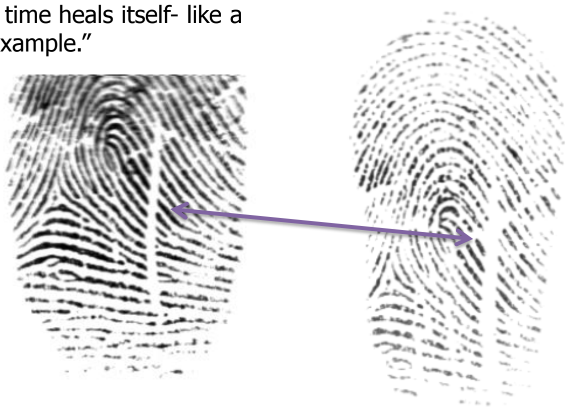

ACE-V Process
Stage 1- Analysis:

Information gathering…" Essentially you are looking for what information you can find within the finger marks."

Pattern Type- Whorl

Core

Delta

Distance between core and delta

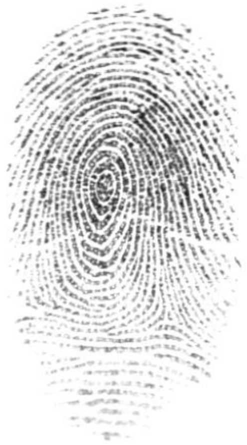

Ridge Enings

Bifurcations

Creases/ Scars

Third level detail

ACE-V Process
Stage 2- Comparison

" So, using the information I have found during the analysis stage I am going to take that through to comparison and actually undertake the comparison."

"I am going to be looking for either very obvious dissimilarities, or start to find similarities."

First Level Detail

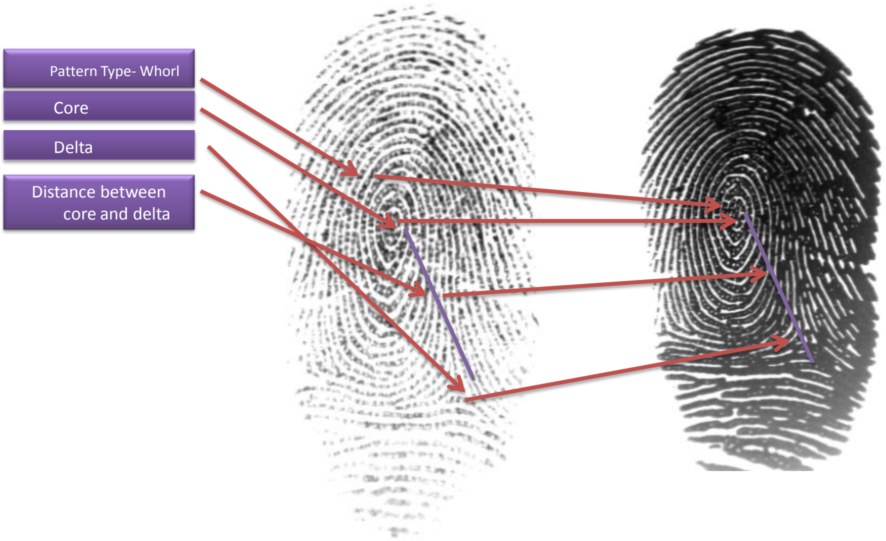

Second Level Detail

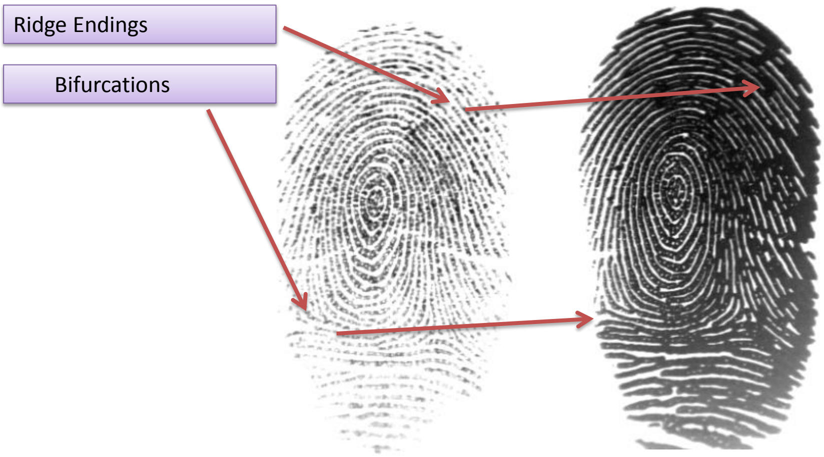

Scars and Creases

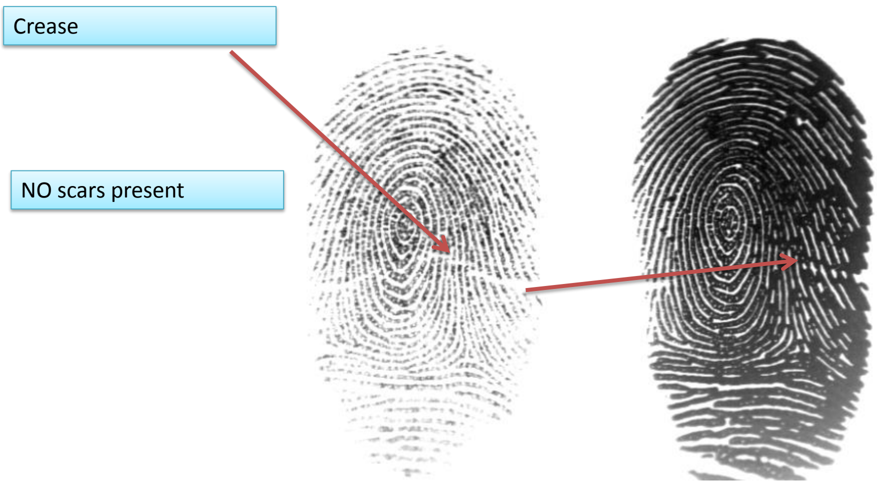

Third Level Detail

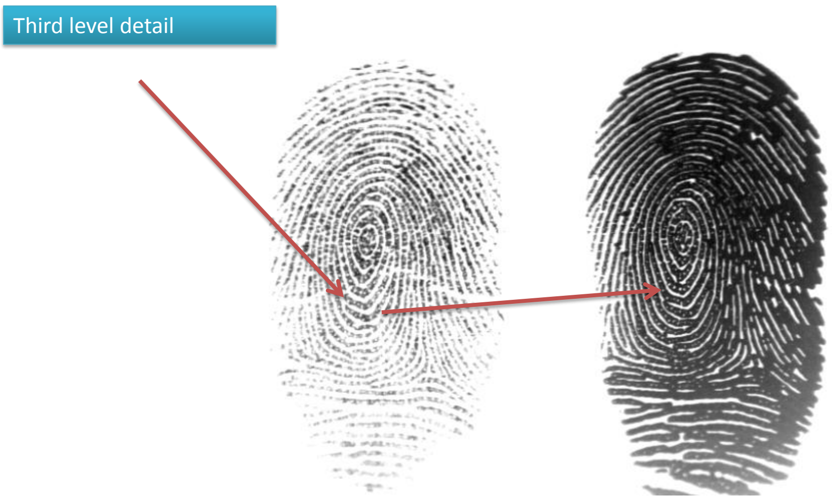

ACE-V Process
Stage 3- Evaluation

"So basically you trying to find … as many details as you're happy with to reach the decision as to whether it's identified or not. So it's all qualitative and quantitative so for a really really good quality crime scene mark, you're not gonna need as many of those features to reach a decision as to whether or not it's identified.

If the crime scene mark is not that good and there's not a lot of detail then you would need a lot more information to reach your decision. A lot of the time it's down to the quality of the impression. "
